# Supplementary material for: Real-world data from a molecular tumor board demonstrates improved outcomes with a precision N-of-One strategy
Source: Nat Commun. 2020 Oct 2;11:4965. doi: 10.1038/s41467-020-18613-3 (PMC7532150; doi:10.1038/s41467-020-18613-3)
Supplement: Supplementary file 1 — Supplementary Information [file 41467_2020_18613_MOESM1_ESM.pdf]

Real-World Data from a Molecular Tumor Board Demonstrates Improved Outcomes with a Precision N-of-One Strategy

Kato et al.

SUPPLEMENTARY INFORMATION

SUPPLEMENTARY METHODS

Matching Scores:

Considerations in evaluating Matching Scores were as follows: (i) if two drugs simultaneously impacted the same aberration and had well-established synergistic effects (e.g., the FDA-approved combinations of trastuzumab and pertuzumab for *ERBB2* alterations or dabrafenib and trametinib for *BRAF* mutations), the impact was counted twice in the numerator and in the denominator; (ii) if a cancer harbored two genomic abnormalities that were in the same gene and had the same biologic effect, these aberrations were counted as one; (iii) two abnormalities in the same gene that were different structurally (e.g., amplification and mutation) and had presumed distinct oncogenic impacts were counted as two; and (iv) androgen receptor-positive or estrogen receptor-positive expression by immunohistochemistry (IHC) targeted by a hormone modulator (e.g., fulvestrant) was also counted as one in both the numerator and denominator.

Antibodies were considered matched if their main target was the product of the molecular alteration. For small molecule inhibitors, matching was based on low inhibitory concentration 50% (IC50) of the drug for the target (<100 nM) or for effectors immediately downstream of the gene product altered. Platinum agents or PARP inhibitors were considered matched if they had a *BRCA*-family alteration. If a patient was given checkpoint inhibitor immunotherapy, the score was assigned 100% for results of high-positive programmed death ligand 1 (PD-L1) by IHC, microsatellite instability-high (MSI-High), and/or high tumor mutational burden (TMB); a score of 50% was assigned for low-positive PD-L1 on IHC and/or TMB-intermediate. No match was scored >100%. Patients in the immunotherapy-treated group who had, as an example,

TMB intermediate and were scored at 50%, and also received matched targeted agents, had the total score calculated as 50% plus (50/100)([number of alterations targeted by drugs administered]/[total number of alterations] (e.g., if tumor had intermediate TMB and received a checkpoint inhibitor, but also had an *ROS1* and *KRAS* alteration and received a *ROS1* inhibitor in addition to checkpoint blockade, the score was 50% plus [50/100] [1/2] = 50% plus 25% = 75%). We also considered *TP53* alterations as matched to anti-VEGF/VEGFR drugs since several publications have shown that *TP53* mutations correlate with upregulation of VEGFA, and that anti-VEGF/VEGFR agents improved treatment outcomes in patients whose tumors harbored *TP53* alterations. [32-36]

We stratified patients according to matching scores ≥50% versus <50% similar to our previous publication. [15]

Stratification of MTB recommendations and treatment assignment

1. If all drugs recommended by MTB as a combination regimen were given, it was considered that physicians complied completely with MTB discussion. Similarly if MTB recommended specific mono therapies (rather than combinations) and the physician gave that mono therapy, it was considered that the physician fully complied.

2. If a combination of drugs was recommended and the doctor gave part but not all of the combination, it was considered that the physician partially complied with the MTB discussion.

3. If none of the drugs given by the doctor were recommended by the MTB, it was considered that the physician did not comply.

Dosing drug combinations

When combinations of drugs were suggested at the MTB, PharmD recommendations and safety rules from previous studies were followed to reduce the drug-related adverse events. [37-39] Most combinations were initiated at ~50% of the standard dose if two drugs were given and at about one-third of dosage if $\geq$3 drugs were combined. The treating physician monitored patients closely, and if the combination therapies were tolerable, dosage was escalated to tolerance.

Supplementary Table 1. Molecular Diagnostic Tests (N=715 unique cases)*

**Tissue NGS/CISH/FISH** [a]	
Foundation One	613
UCSD Comprehensive NGS Solid Tumor Mutation Panel Analysis	25
Paradigm NGS	21
Copy number variation	11
Gene fusion	9
Omniseq NGS	19
Caris NGS	21
CISH	10
FISH	7
Tempus NGS	6
Cancer Genetics Inc. NGS	4
FISH	3
**Cell-free circulating tumor DNA** [b]	
Guardant	271
Foundation ACT	50
**mRNA expression analysis** [c]	
Paradigm	25
Cancer Genetics	11
Caris	5
**Immunohistochemistry** [d]	
Caris	86
Paradigm	30
UCSD	4
Nantomics	3
**Targets associated with immunotherapy (TMB, MSI, PD-L1)** [e]	
Foundation One	295
Caris	70
Paradigm	38
Omniseq	19
UCSD	14
Tempus	5
Nantomics	4
Cancer Genetics Inc.	2

[a] 646 patients had 742 tissue NGS/CISH/FISH evaluations.
[b] 309 patients had 321 cell-free circulating tumor DNA evaluations.
[c] 39 patients had 41 mRNA expression analysis.

[d] 115 patients had 120 immunohistochemistry evaluations.
[e] 362 patients had 446 tests for targets associated with immunotherapy.
Abbreviation: CISH, chromogenic in situ hybridization; FISH, fluorescence in situ hybridization; NGS, next-generation sequencing; TMB, tumor mutational burden; UCSD, University of California San Diego

*Websites of the testing laboratory:
Cancer Genetics Incorporated, https://www.cancergenetics.com/;
Caris Life Sciences, https://www.carismolecularintelligence.com/molecular-testing-services/;
Foundation One and Foundation ACT, https://www.foundationmedicine.com/;
Guardant360, http://www.guardant360.com/;
Nantomics, https://nantomics.com/; OmniSeq, https://www.omniseq.com/;
Paradigm Diagnostics, https://www.paradigmdx.com/;
Tempus, https://www.tempus.com/genomic-sequencing/;
If a patient had two or more tests from the same testing laboratory, the sample closest to the time of MTB was used for the analysis.

Supplementary Table 2. Frequency of characterized genomic alterations by tissue NGS (N=646). (See **Figure 1.A.**)

	Any alterations N (%)		Mutation N (%)		Rearrangement N (%)		Deletion N (%)		Amplification N (%)		Insertion N (%)		Multiple alterations N (%)	
TP53	338	(52.3%)	298	(46.1%)	0	(0.0%)	10	(1.5%)	1	(0.2%)	0	(0.0%)	29	(4.5%)
KRAS	154	(23.8%)	140	(21.7%)	0	(0.0%)	0	(0.0%)	11	(1.7%)	0	(0.0%)	3	(0.5%)
PIK3CA	102	(15.8%)	79	(12.2%)	0	(0.0%)	1	(0.2%)	11	(1.7%)	0	(0.0%)	11	(1.7%)
APC	88	(13.6%)	24	(3.7%)	0	(0.0%)	5	(0.8%)	0	(0.0%)	26	(4.0%)	33	(5.1%)
CDKN2A/B	74	(11.5%)	0	(0.0%)	0	(0.0%)	71	(11.0%)	0	(0.0%)	0	(0.0%)	3	(0.5%)
MYC	72	(11.1%)	1	(0.2%)	2	(0.3%)	0	(0.0%)	68	(10.5%)	1	(0.2%)	0	(0.0%)
ERBB2	56	(8.7%)	14	(2.2%)	0	(0.0%)	0	(0.0%)	34	(5.3%)	3	(0.5%)	5	(0.8%)
PTEN	55	(8.5%)	33	(5.1%)	0	(0.0%)	19	(2.9%)	0	(0.0%)	0	(0.0%)	3	(0.5%)
CDKN2A	54	(8.4%)	13	(2.0%)	0	(0.0%)	11	(1.7%)	0	(0.0%)	6	(0.9%)	24	(3.7%)
SMAD4	53	(8.2%)	34	(5.3%)	0	(0.0%)	17	(2.6%)	1	(0.2%)	0	(0.0%)	1	(0.2%)
CCND1	52	(8.0%)	2	(0.3%)	0	(0.0%)	0	(0.0%)	50	(7.7%)	0	(0.0%)	0	(0.0%)
ARID1A	47	(7.3%)	15	(2.3%)	0	(0.0%)	4	(0.6%)	1	(0.2%)	25	(3.9%)	2	(0.3%)
FGF19	45	(7.0%)	0	(0.0%)	0	(0.0%)	0	(0.0%)	45	(7.0%)	0	(0.0%)	0	(0.0%)
FGF4	44	(6.8%)	0	(0.0%)	0	(0.0%)	0	(0.0%)	44	(6.8%)	0	(0.0%)	0	(0.0%)
BRCA2	42	(6.5%)	17	(2.6%)	0	(0.0%)	3	(0.5%)	0	(0.0%)	16	(2.5%)	6	(0.9%)
FGF3	42	(6.5%)	0	(0.0%)	0	(0.0%)	0	(0.0%)	42	(6.5%)	0	(0.0%)	0	(0.0%)
RB1	40	(6.2%)	18	(2.8%)	1	(0.2%)	17	(2.6%)	0	(0.0%)	1	(0.2%)	3	(0.5%)

TERT	36	(5.6%)	35	(5.4%)	0	(0.0%)	0	(0.0%)	1	(0.2%)	0	(0.0%)	0	(0.0%)
GNAS	33	(5.1%)	18	(2.8%)	0	(0.0%)	0	(0.0%)	13	(2.0%)	1	(0.2%)	1	(0.2%)
MLL2	33	(5.1%)	24	(3.7%)	0	(0.0%)	1	(0.2%)	0	(0.0%)	0	(0.0%)	8	(1.2%)
FBXW7	31	(4.8%)	27	(4.2%)	0	(0.0%)	2	(0.3%)	0	(0.0%)	0	(0.0%)	2	(0.3%)
FGFR1	28	(4.3%)	4	(0.6%)	0	(0.0%)	0	(0.0%)	24	(3.7%)	0	(0.0%)	0	(0.0%)
ZNF217	28	(4.3%)	0	(0.0%)	0	(0.0%)	0	(0.0%)	28	(4.3%)	0	(0.0%)	0	(0.0%)
BRAF	27	(4.2%)	23	(3.6%)	1	(0.2%)	2	(0.3%)	0	(0.0%)	0	(0.0%)	1	(0.2%)
MDM2	27	(4.2%)	0	(0.0%)	0	(0.0%)	0	(0.0%)	27	(4.2%)	0	(0.0%)	0	(0.0%)
NF1	27	(4.2%)	13	(2.0%)	2	(0.3%)	7	(1.1%)	1	(0.2%)	0	(0.0%)	4	(0.6%)
NOTCH1	23	(3.6%)	18	(2.8%)	1	(0.2%)	3	(0.5%)	0	(0.0%)	0	(0.0%)	1	(0.2%)
EGFR	22	(3.4%)	5	(0.8%)	0	(0.0%)	0	(0.0%)	11	(1.7%)	1	(0.2%)	5	(0.8%)
CDK4	21	(3.3%)	2	(0.3%)	0	(0.0%)	0	(0.0%)	19	(2.9%)	0	(0.0%)	0	(0.0%)
ZNF703	21	(3.3%)	0	(0.0%)	0	(0.0%)	0	(0.0%)	21	(3.3%)	0	(0.0%)	0	(0.0%)

Alterations with >3% were included.

Supplementary Table 3. Frequency of characterized genomic alterations by cfDNA (N=309) (See **Figure 1.B.**)

	Any alterations N (%)		Mutation N (%)		Rearrangement N (%)		Deletion N (%)		Amplification N (%)		Insertion N (%)		Multiple alterations N (%)	
TP53	151	(48.9%)	121	(39.2%)	0	(0.0%)	1	(0.3%)	0	(0.0%)	0	(0.0%)	29	(9.4%)
KRAS	69	(22.3%)	49	(15.9%)	0	(0.0%)	0	(0.0%)	11	(3.6%)	0	(0.0%)	9	(2.9%)
PIK3CA	57	(18.4%)	28	(9.1%)	0	(0.0%)	0	(0.0%)	19	(6.1%)	1	(0.3%)	9	(2.9%)
BRAF	38	(12.3%)	13	(4.2%)	0	(0.0%)	0	(0.0%)	24	(7.8%)	0	(0.0%)	1	(0.3%)
EGFR	37	(12.0%)	7	(2.3%)	0	(0.0%)	2	(0.6%)	24	(7.8%)	0	(0.0%)	4	(1.3%)
MYC	33	(10.7%)	1	(0.3%)	0	(0.0%)	0	(0.0%)	32	(10.4%)	0	(0.0%)	0	(0.0%)
APC	31	(10.0%)	27	(8.7%)	0	(0.0%)	1	(0.3%)	0	(0.0%)	0	(0.0%)	3	(1.0%)
CDK6	26	(8.4%)	3	(1.0%)	0	(0.0%)	0	(0.0%)	22	(7.1%)	0	(0.0%)	1	(0.3%)
MET	23	(7.4%)	4	(1.3%)	0	(0.0%)	0	(0.0%)	19	(6.1%)	0	(0.0%)	0	(0.0%)
FGFR1	22	(7.1%)	4	(1.3%)	0	(0.0%)	0	(0.0%)	17	(5.5%)	0	(0.0%)	1	(0.3%)
KIT	22	(7.1%)	8	(2.6%)	0	(0.0%)	0	(0.0%)	11	(3.6%)	0	(0.0%)	3	(1.0%)
PDGFRA	22	(7.1%)	10	(3.2%)	0	(0.0%)	0	(0.0%)	10	(3.2%)	0	(0.0%)	2	(0.6%)
ERBB2	21	(6.8%)	9	(2.9%)	0	(0.0%)	0	(0.0%)	11	(3.6%)	0	(0.0%)	1	(0.3%)
CCNE1	18	(5.8%)	1	(0.3%)	0	(0.0%)	0	(0.0%)	17	(5.5%)	0	(0.0%)	0	(0.0%)
AR	16	(5.2%)	7	(2.3%)	0	(0.0%)	0	(0.0%)	9	(2.9%)	0	(0.0%)	0	(0.0%)
ARID1A	15	(4.9%)	12	(3.9%)	0	(0.0%)	0	(0.0%)	0	(0.0%)	1	(0.3%)	2	(0.6%)
NF1	15	(4.9%)	12	(3.9%)	0	(0.0%)	0	(0.0%)	0	(0.0%)	0	(0.0%)	3	(1.0%)
RAF1	15	(4.9%)	5	(1.6%)	0	(0.0%)	0	(0.0%)	9	(2.9%)	0	(0.0%)	1	(0.3%)
CCND1	13	(4.2%)	3	(1.0%)	0	(0.0%)	0	(0.0%)	10	(3.2%)	0	(0.0%)	0	(0.0%)
PTEN	13	(4.2%)	13	(4.2%)	0	(0.0%)	0	(0.0%)	0	(0.0%)	0	(0.0%)	0	(0.0%)

CCND2	12	(3.9%)	5	(1.6%)	0	(0.0%)	0	(0.0%)	7	(2.3%)	0	(0.0%)	0	(0.0%)
CDKN2A	12	(3.9%)	12	(3.9%)	0	(0.0%)	0	(0.0%)	0	(0.0%)	0	(0.0%)	0	(0.0%)
ESR1	12	(3.9%)	8	(2.6%)	0	(0.0%)	0	(0.0%)	1	(0.3%)	0	(0.0%)	3	(1.0%)
BRCA2	11	(3.6%)	7	(2.3%)	0	(0.0%)	0	(0.0%)	0	(0.0%)	0	(0.0%)	4	(1.3%)
GNAS	11	(3.6%)	11	(3.6%)	0	(0.0%)	0	(0.0%)	0	(0.0%)	0	(0.0%)	0	(0.0%)
SMAD4	10	(3.2%)	9	(2.9%)	0	(0.0%)	0	(0.0%)	0	(0.0%)	0	(0.0%)	1	(0.3%)
CDK4	8	(2.6%)	1	(0.3%)	0	(0.0%)	0	(0.0%)	7	(2.3%)	0	(0.0%)	0	(0.0%)
FGFR2	8	(2.6%)	6	(1.9%)	0	(0.0%)	0	(0.0%)	2	(0.6%)	0	(0.0%)	0	(0.0%)
FGFR3	8	(2.6%)	5	(1.6%)	2	(0.6%)	0	(0.0%)	0	(0.0%)	0	(0.0%)	1	(0.3%)
NOTCH1	8	(2.6%)	7	(2.3%)	0	(0.0%)	0	(0.0%)	0	(0.0%)	0	(0.0%)	1	(0.3%)

Alterations with >2.5% were included.

Supplementary Table 4. Number and percentage of patients with pertinent protein markers detected by IHC (N=115)

Pertinent protein abnormalities	Number of patients with affected protein/Number of patients tested	Implications of protein markers
RRM1 (-)	33 / 40 (83%)	Low or loss of RRM1 (ribonucleotide reductase catalytic subunit M1) is potentially targetable with gemcitabine.
TOP2A (+)	29 / 37 (78%)	Positive TOP2A (topoisomerase II alpha) predicts the response to topoisomerase II inhibitor such as doxorubicin.
ERCC1 (-)	37 / 52 (71%)	Loss of ERCC1 (excision repair cross-complementation group 1) is potential marker for response to platinum.
TOPO1 (+)	61 / 89 (69%)	Positive TOPO1 (topoisomerase I) is potential marker for response to topoisomerase I inhibitors such as irinotecan or topotecan.
TS (-)	52 / 81 (64%)	Negative TS (thymidylate synthase) is potentially targetable with 5-fluorouracil.
TUBB3 (-)	34 / 59 (58%)	Negative TUBB3 (class III beta-tubulin) is predictive marker for response with taxane-based therapy.
TLE3 (+)	23 / 40 (58%)	Positive TLE3 (transducin like enhancer of split 3) is potential marker for response to taxane therapy.
MGMT (-)	25 / 57 (44%)	Negative MGMT (O(6)-methylguanine-DNA methyltransferase) may predict response to alkylating agents such as dacarbazine.
cMET (+)	13 / 34 (38%)	Positive cMET is potential marker for response to Met inhibitors
SPARC Polyclonal (+)	6 / 20 (30%)	Positive SPARC (Secreted Protein Acidic and Rich in Cysteine) is potential marker for response to taxanes.

PTEN (-)	17 / 60 (28%)	PTEN (phosphatase and tensin homolog) loss is potentially targetable with mTOR inhibitors.
ER (+)	10 / 49 (20%)	Positive ER (estrogen receptor) predicts response to hormone modulator.
SPARC Monoclonal (+)	2 / 10 (20%)	Positive SPARC (Secreted Protein Acidic and Rich in Cysteine) is potential marker for response to taxanes.
AR (+)	7 / 40 (18%)	Positive AR (androgen receptor) predicts response to hormone modulator.
PR (+)	3 / 48 (6%)	Positive PR (progesterone receptor) predicts response to hormone modulator.
HER2 (+)**	3 / 67 (4%)	Positive HER2 (human epidermal growth factor receptor 2) is potential marker for response to HER2 inhibitors.
ALK (+)	0 / 41 (0%)	Positive ALK (anaplastic lymphoma kinase) is potential marker for response to ALK inhibitor.

Criteria for positivity*

**Pertinent protein abnormalities**	**Results of IHC considered actionable**	**Condition for positive IHC results**
**RRM1**	Negative	Intensity ≥2+ and ≥50% of cells stained
**TOP2A**	Positive	Intensity ≥1+ and ≥10% of cells stained
**ERCC1**	Negative	Intensity of ≥3+ with ≥10% or ≥2+ with ≥50% of cells stained
**TOPO1**	Positive	Intensity ≥2+ and ≥30% of cells stained
**TS**	Negative	Intensity ≥1+ and ≥10% of cells stained
**TUBB3**	Negative	Intensity ≥2+ and ≥30% of cells stained
**TLE3**	Positive	Intensity ≥2+ and ≥30% of cells stained
**MGMT**	Negative	Intensity ≥1+ and >35% of cells stained

**PTEN**	Negative	Intensity ≥1+ and >50% of cells stained
**ER****	Positive	Intensity ≥1+ and ≥10% of cells stained
**EGFR**	Positive	Intensity ≥1+ and ≥10% of cells stained
**AR****	Positive	Intensity ≥1+ and ≥10% of cells stained
**PR**	Positive	Intensity ≥1+ and ≥10% of cells stained

*Criteria for selected positive IHC results according to Caris Life Sciences (www.carismolecularintelligence.com).
** Positivity consistent with NCCN guidelines

Abbreviations:
AR, androgen receptor; ER, estrogen receptor; ERCC1, excision repair cross-complementation group 1; MGMT, O(6)-methylguanine-DNA methyltransferase; PR, progesterone receptor; PTEN, phosphatase and tensin homolog; RRM1, ribonucleotide reductase catalytic subunit M1; TLE3, transducin like enhancer of split 3; TOPO1, topoisomerase I; TOP2A, topoisomerase II alpha; TS, thymidylate synthase; TUBB3, class III beta-tubulin.

Supplementary Table 5. mRNA expression level among patients with diverse cancers (N=39 had selected mRNA profiling)*

	Low expression: N (%)		High expression N (%)		Total number of patients being tested
ERCC1	11	73.3%	4	26.7%	15
TUBB3	1	6.7%	14	93.3%	15
TS	8	61.5%	5	38.5%	13
AREG	1	7.7%	12	92.3%	13
BAX	0	0%	13	100.0%	13
TOP2A	2	16.7%	10	83.3%	12
BIRC5	0	0%	11	100%	11
MET	0	0%	11	100%	11
PTEN	10	100.0%	0	0.0%	10
BRCA1	1	10.0%	9	90.0%	10
CA IX	0	0%	10	100%	10
VEGFR2	0	0%	10	100%	10
ERBB2	0	0%	9	100%	9
TYMS	0	0%	9	100%	9
ESR1	0	0%	8	100%	8
EGFR	4	57.1%	3	42.9%	7
EREG	1	14.3%	6	85.7%	7
ERBB3	0	0%	7	100%	7
IGF1R	0	0%	7	100%	7
TYMP	0	0%	7	100%	7
CES2	0	0%	6	100%	6
KIT	0	0%	6	100%	6
LRP6	0	0%	6	100%	6

NFKB1	0	0%	6	100%	6
PDGFRB	0	0%	6	100%	6
RelA	0	0%	6	100%	6
EPHA2	0	0%	5	100%	5
EZH2	0	0%	5	100%	5
SLC29A1	0	0%	5	100%	5
RRM1	1	25.0%	3	75.0%	4

* Included when ≥4 cases were evaluated for the specific mRNA markers.

See **Supplementary Table 4** for potential actionability of the selected markers.

Most mRNA expression profiling was done at Paradigm Diagnostics. High mRNA expression was defined as > 5 fold change in expression as compared to a set of normal tissue controls (at least 6 normal tissue control from ~30 different primary tumor types) (https://www.paradigmdx.com/).

Supplementary Table 6. Association between Matching Score and therapies after MTB discussion.

Treatment course after the Molecular Tumor Board discussion	Matching Score <50% N (%)		Matching Score ≥50% N (%)		Total N
**Received all recommended medications**	38	(44.2%)	48	(55.8%)	86
**Received part of recommended medications**	109	(60.9%)	70	(39.1%)	179
**Received physician's choice regimen**	157	(95.7%)	7	(4.3%)	164

Supplementary Figure 1. CONSORT diagram of patients included in the MTB study*

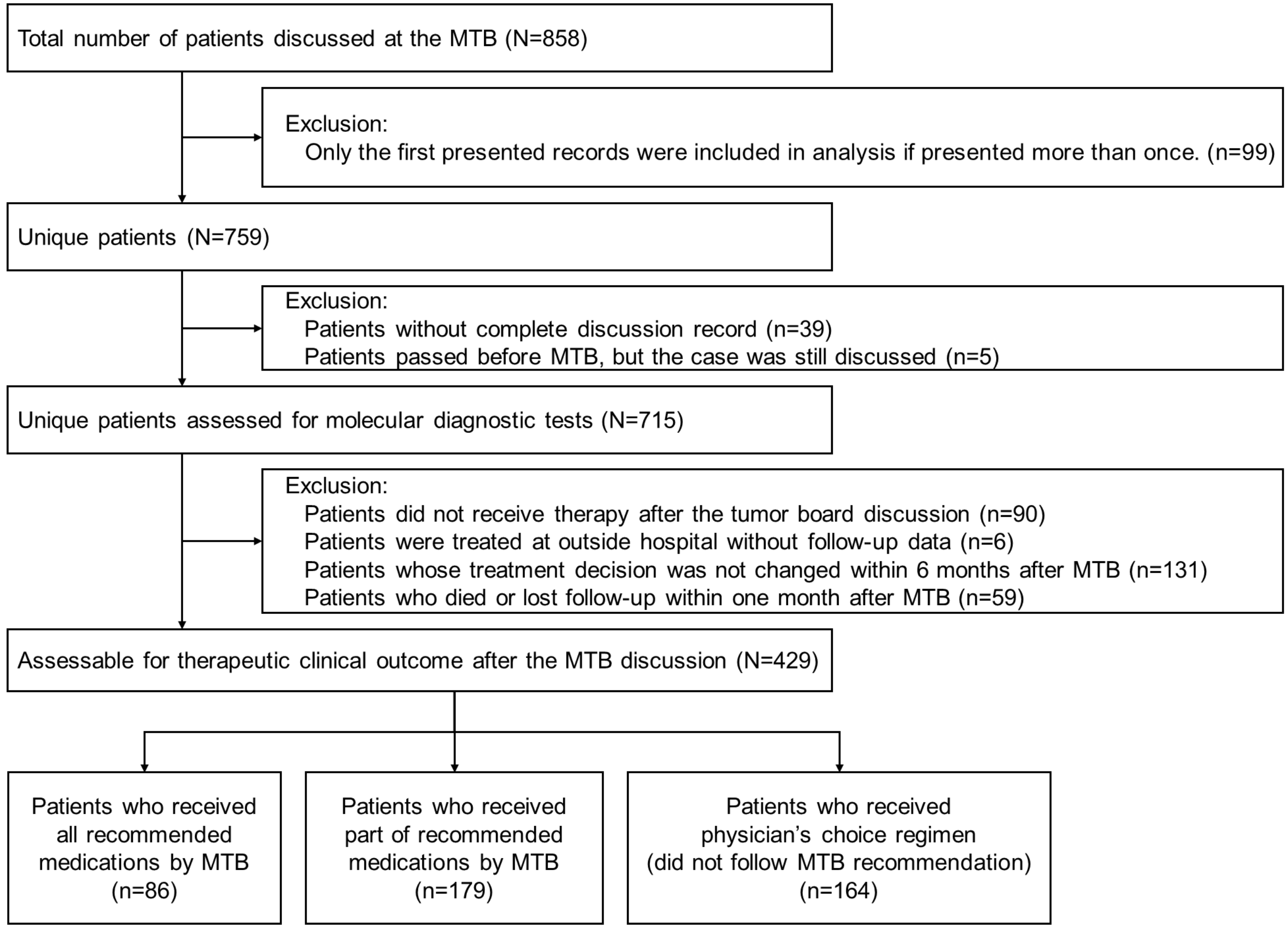

*Twenty-seven of 265 patients with matched therapy were included in the IPREDICT study with 73 matched patients [15]; other patients were not included because IPREDICT patients were often presented in a protocol-specific special electronic MTB, the results of which were not tabulated herein (only face-to-face MTB was included here).

Supplementary Figure 2. Frequency of potentially actionable protein markers (N=115) (See **Supplementary Table 4**)

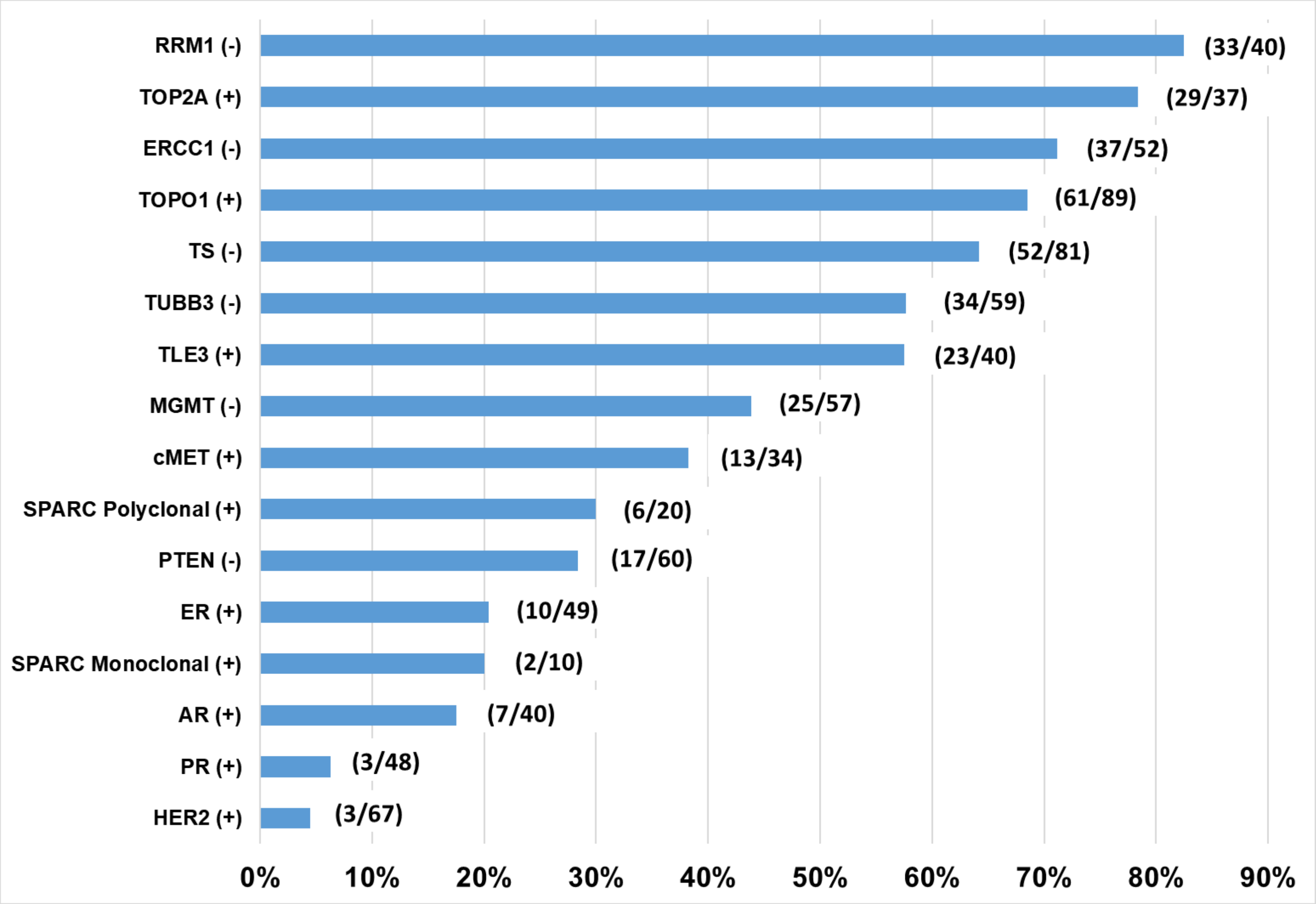

* Percent indicates percent of the patient with pertinent IHC results with pertinent protein marker for targeted approach. Presented when protein markers were included in MTB agenda (Supplementary Table 1).
