# Supplementary material for: Real-world data from a molecular tumor board demonstrates improved outcomes with a precision N-of-One strategy
Source: Nat Commun. 2020 Oct 2;11:4965. doi: 10.1038/s41467-020-18613-3 (PMC7532150; doi:10.1038/s41467-020-18613-3)
Supplement: Supplementary file 3 — Description of Additional Supplementary Files [file 41467_2020_18613_MOESM3_ESM.pdf]

Description of Additional Supplementary Files

Title: Supplementary Dataset 1

Description: Clinical characteristics of 429 patients who were evaluable for therapeutic outcome after MTB discussion
