# Supplementary material for: Real-world data from a molecular tumor board demonstrates improved outcomes with a precision N-of-One strategy
Source: Nat Commun. 2020 Oct 2;11:4965. doi: 10.1038/s41467-020-18613-3 (PMC7532150; doi:10.1038/s41467-020-18613-3)
Supplement: Supplementary file 5 — Reporting Summary [file 41467_2020_18613_MOESM5_ESM.pdf]

nature research

Corresponding author(s): Shumei Kato

Last updated by author(s): 2020/08/18

Reporting Summary

Nature Research wishes to improve the reproducibility of the work that we publish. This form provides structure for consistency and transparency in reporting. For further information on Nature Research policies, see our Editorial Policies and the Editorial Policy Checklist.

Statistics

For all statistical analyses, confirm that the following items are present in the figure legend, table legend, main text, or Methods section.

n/a	Confirmed	
☐	☒	The exact sample size (n) for each experimental group/condition, given as a discrete number and unit of measurement
☒	☐	A statement on whether measurements were taken from distinct samples or whether the same sample was measured repeatedly
☐	☒	The statistical test(s) used AND whether they are one- or two-sided<br>*Only common tests should be described solely by name; describe more complex techniques in the Methods section.*
☐	☒	A description of all covariates tested
☐	☒	A description of any assumptions or corrections, such as tests of normality and adjustment for multiple comparisons
☐	☒	A full description of the statistical parameters including central tendency (e.g. means) or other basic estimates (e.g. regression coefficient) AND variation (e.g. standard deviation) or associated estimates of uncertainty (e.g. confidence intervals)
☐	☒	For null hypothesis testing, the test statistic (e.g. F, t, r) with confidence intervals, effect sizes, degrees of freedom and P value noted<br>*Give P values as exact values whenever suitable.*
☒	☐	For Bayesian analysis, information on the choice of priors and Markov chain Monte Carlo settings
☒	☐	For hierarchical and complex designs, identification of the appropriate level for tests and full reporting of outcomes
☒	☐	Estimates of effect sizes (e.g. Cohen's d, Pearson's r), indicating how they were calculated

Our web collection on statistics for biologists contains articles on many of the points above.

Software and code

Policy information about availability of computer code

Data collection	No software used for the data collection
Data analysis	SPSS, version 25 (IBM Corporation, Armonk, NY).

For manuscripts utilizing custom algorithms or software that are central to the research but not yet described in published literature, software must be made available to editors and reviewers. We strongly encourage code deposition in a community repository (e.g. GitHub). See the Nature Research guidelines for submitting code & software for further information.

Data

Policy information about availability of data

All manuscripts must include a data availability statement. This statement should provide the following information, where applicable:

- Accession codes, unique identifiers, or web links for publicly available datasets
- A list of figures that have associated raw data
- A description of any restrictions on data availability

All data generated or analyzed during this study are included in this published article (and its supplementary information files).

Field-specific reporting

Life sciences study design

All studies must disclose on these points even when the disclosure is negative.

Sample size	This was observation study and the sample size was based on the patients presented at the Molecular Tumor Board at UCSD during December 2012 and September 2018.
Data exclusions	All patients that were discussed at the Molecular Tumor Board were included. For the analysis of therapeutic clinical outcome, we have included the patients who were evaluable for the assessment for the treatment outcome (this was not pre-established)
Replication	Replication was not feasible in this study. This is an observation study among patients who were presented at the Molecular Tumor Board and it is an unique patient population.
Randomization	Randomization was not feasible since this is an observation study.
Blinding	Blinding was not feasible since this is an observation study.

Reporting for specific materials, systems and methods

We require information from authors about some types of materials, experimental systems and methods used in many studies. Here, indicate whether each material, system or method listed is relevant to your study. If you are not sure if a list item applies to your research, read the appropriate section before selecting a response.

Materials & experimental systems

n/a	Involved in the study	
☒	☐	Antibodies
☒	☐	Eukaryotic cell lines
☒	☐	Palaeontology and archaeology
☒	☐	Animals and other organisms
☐	☒	Human research participants
☐	☒	Clinical data
☒	☐	Dual use research of concern

Methods

n/a	Involved in the study	
☒	☐	ChIP-seq
☒	☐	Flow cytometry
☒	☐	MRI-based neuroimaging

Human research participants

Policy information about studies involving human research participants

Population characteristics	Patients with diverse cancer types (most commonly with breast cancer [18%], followed by colorectal [12.2%], hematologic and gastroesophageal cancer [7.1%]) with median age of 61 years old. 58.7% were woman.
Recruitment	Patients were recruited at the UCSD Cancer Center through clinic visit. The recruitment was based on physician's decision, which may have lead to selection bias.
Ethics oversight	The study was approved by UCSD Institutional Review Board. Informed consent was obtained from legally acceptable representative.

Note that full information on the approval of the study protocol must also be provided in the manuscript.

Clinical data

Policy information about clinical studies

All manuscripts should comply with the ICMJEguidelines for publication of clinical research and a completedCONSORT checklist must be included with all submissions.

Clinical trial registration	NCT02478931
Study protocol	https://clinicaltrials.gov/ct2/show/NCT02478931
Data collection	Data was collected at Moores UCSD Cancer Center.
Outcomes	Clinical outcomes (progression-free survival, overall survival and response) that were not pre-defined were assessed using Log-rank test and Kaplan-Meier analysis. Due to the nature of observation study, there is no pre-defined primary and secondary endpoint.
